# Immunosuppression and stem cell stimulation to treat AA: clinical and biologic implications

**DOI:** 10.1097/HS9.0000000000000211

**Published:** 2019-06-30

**Authors:** Neal S. Young

**Affiliations:** National Heart, Lung, and Blood Institute, NIH, Bethesda, United States

**Take home messages**

- Understand classification and pathophysiology of immune AA.
- Be aware of current outcome data with standard medical therapy of severe AA.
- Appreciate basic science implications of clinical observations.

## Introduction

Low peripheral blood counts can occur in a variety of clinical circumstances, secondary to infections, malignancies, and systemic diseases. Pancytopenia with a hypocellular marrow has a more restricted differential diagnosis. Historical “aplastic anemia” (AA) has 3 pathophysiologies: damage, secondary to chemicals, radiation, and some medical drugs (benzene poisoning the classic syndrome); in multiorgan genetic syndromes (Fanconi anemia the paradigm); and immune-mediated.^1▪^ In the modern era, immune AA is most often the diagnosis in the young patient with sudden onset of severe pancytopenia and a hypocellular bone marrow.

In immune AA, the response of patients to therapy has been highly informative of underlying pathological processes.^2^^,^^3▪^ AA was one of the first diseases to be cured by bone marrow transplant, an important demonstration of the hematopoietic stem cell in humans. Graft rejection and graft-versus-host disease have been characterized in AA without the complicating features often present when hematologic malignancies are the indication for transplant.

Nontransplant therapies also are effective in immune AA.^4▪^ Improvement of blood counts after conditioning with antilymphocyte globulin, in the setting of a failed stem cell transplant, first suggested an immune basis for destruction of hematopoietic stem and progenitor cells. The observation of incidental autologous reconstitution was exploited in purposeful trials, first of antithymocyte globulins (ATG) and later with a variety of immunomodulatory biologics and drugs.

## Current state of the art

### Immunosuppression for immune AA

ATGs produced hematologic responses in about 50% of patients. The first improvement on ATG therapy was addition of cyclosporine; cyclosporine was assessed empirically, but also with the motive of blockade of T cell activation concurrent with depletion of lymphocytes by polyclonal antibodies. Cyclosporine combined with ATG increased the hematologic response rate to 60% to 65%.^5^,^6^ Cyclosporine alone is only modestly effective. Combined therapy has been standard first-line therapy in severe AA for decades.

Attempts to improve on ATG plus cyclosporine, by addition of androgens, granulocyte colony stimulating factor, mycophenolate, or rapamycin, have not altered response rates or long-term outcomes. Efforts to replace horse ATG with more potent immunosuppressives—rabbit ATG or alemtuzumab or cyclophosphamide^7^—also have failed, due to lower response rates or unacceptable toxicities.^2^

A frequent late complication of immunosuppressive therapy is relapse, declining blood counts usually necessitating reinstitution of cyclosporine or increase in its dose, and cyclosporine-dependence, which occurs in 30% or more patients.^8^ A more serious complication is development of cytogenetic abnormalities in the marrow, sometimes with frank dysplasia and even leukemia, in about 15% of cases over a decade post-ATG. “Clonal evolution,” especially chromosome 7 loss or involvement of multiple chromosomes, has a poor prognosis and is an indication for stem cell transplant.

Responsiveness to immunosuppression is strong evidence for an immune pathophysiology, and clinical results have stimulated experimental laboratory research. Because ATG is directed at thymocytes, lymphocytes, especially cytotoxic T cells, have been the focus, as for example demonstration of oligoclones of activated in patients, which appear to be targets of therapy. Notably, cell populations are small, and while clones decline with ATG administration, they are not eradicated, and new clones may arise over time. These results are consistent with a chronic, often relapsing immune disease, in which destruction of target cells leads to epitope spreading. Immune AA can be modeled in the mouse by infusion of lymphocytes mismatched at major or minor histocompatibility antigens, but the inciting antigens in human disease remain unknown. Mouse models suggest that the cellular immune response may be complex, involving different T cell subsets and macrophages. A large proportion of patients with low-risk myelodysplastic syndrome respond to immune therapy.^9^ While there is increasing interest in the role of innate immunity in marrow failure in MDS, it is also possible that somatically mutated or aneuploid cells are targets of an adaptive immune response, with “innocent bystander” killing of normal hematopoietic cells and selection of resistant clones over time.^10^

### Eltrombopag as stem cell stimulation in bone marrow failure

Hematopoietic growth factors have generally been ineffective in AA.^11^ It was therefore unexpected when eltrombopag, a synthetic mimetic of thrombopoietin, showed activity in patients with refractory AA, about half of whom responded with robust trilineage improvements in blood counts, most durable after discontinuation of drug.^12^ Eltrombopag has been relabeled for this indication. Added to initial standard immunosuppression, eltrombopag increased the overall response rate to about 80% and the complete response rate to about 50%, with patients often showing more rapid than expected hematologic recovery.^13^ To date, the rates of relapse and evolution to myeloid malignancies appear similar or lower than in historical controls treated with immunosuppression alone. Eltrombopag has also shown activity in other marrow failure syndromes, moderate AA, and low-risk MDS.

## Future perspectives

What are some clinical and biologic implications of decades of relative success with immunosuppression in AA? First, we still do not fully understand the mechanism of action of ATG. Cytotoxic T cells are the presumed target, but more potent lymphocyte depletion does not improve the response rate. Horse ATG may better conserve regulatory T cells, beneficial in an immune-mediated disease. Second, there has been little motivation to alter the usual dose or duration of either ATG or cyclosporine, and as a result the optimal regimen is not known. Third, strategies to prevent relapse range from extended cyclosporine administration through tapering dosage to expectant monitoring of blood counts. Fourth, there are no reliable predictors of evolution, although telomere length rather than somatically mutated clones correlate.^14▪^^,^^15▪^

Increased bone marrow cellularity, CD34 cell and progenitor numbers after therapy suggest a direct effect of eltrombopag on marrow stem cells. Thrombopoietin concentrations in the blood of AA patients are very high, but eltrombopag may function by evading a block to receptor engagement in the presence of interferon-γ (Alvarado and Larochelle, personal communication). Eltrombopag may also have other beneficial activities, such as iron chelation and augmentation of regulatory T cells. That older patients and those with lower reticulocyte counts are more likely to benefit from addition of eltrombopag to standard therapy is consistent with stem cell stimulation. Clinical results have indicated that patients with AA may have sufficient stem cell reserves to maintain normal blood counts long term, and the patterns of relapse have implications for stem cell biology. “Relapse” is often only manifest as a lowered platelet count, consistent with the particular relationship of the stem cell and megakaryocyte. Clonal evolution may be less frequent when more stem cells are recruited with eltrombopag. However, the consistent observation of early clonal evolution with monotherapy in refractory patients strong suggests that eltrombopag may directly elicit proliferation of aneuploid cells.
